# Reliance on Pumped Mother’s Milk Has an Environmental Impact

**DOI:** 10.3390/children3030014

**Published:** 2016-09-10

**Authors:** Genevieve Becker, Yvonne Ryan-Fogarty

**Affiliations:** 1 BEST Services, Galway H91T22T, Ireland; 2 Chemical and Environmental Sciences Department, University of Limerick, Limerick V94 T9PX, Ireland; yvonne.ryan@ul.ie

**Keywords:** human milk, breast pump, environmental impact, green healthcare, life cycle analysis

## Abstract

Breastfeeding is an environmentally friendly process; however when feeding relies on pumped mother’s milk, the environmental picture changes. Waste plastics and heavy metals raise concerns regarding resource efficiency, waste treatment, and detrimental effects on health. Reliance on pumped milk rather than breastfeeding may also effect obesity and family size, which in turn have further environmental impacts. Information on pump equipment rarely includes environmental information and may focus on marketing the product for maximum profit. In order for parents, health workers, and health policy makers to make informed decisions about the reliance on pumped mother’s milk, they need information on the broad and far reaching environmental aspects. There was no published research found that examined the environmental impact of using pumped mother’s milk. A project is ongoing to examine this issue.

Breastfeeding is acknowledged as environmentally friendly as it “generally requires no containers, no paper or plastic, no fuel to prepare, and no transportation to deliver” [1]. However, when this feeding relies on pumped milk, the environmental picture changes. Two separate research activities undertook literature searches on the broader topics of the methods of milk expression [2] and the environmental aspects of infant feeding [3], which would have been expected to find studies on the environmental impact of pumping milk. No such published research was found. A specific literature search was also carried out (30 July 2016) for the purpose of this article in the databases for Web of Science including the Science Citation Index, Social Sciences Citation Index, and Arts & Humanities Citation Index; Scopus; Science Direct; Proquest Dissertations and Theses: Abstracts & Index; EMBASE; and Environment Complete, using the terms “milk”, “pump” or “express”, “environment” or “waste”, and “human” appearing in the title or abstract with no date limits. No relevant literature was found in this search.

The total environmental impact of pumping milk is currently unknown and merits inclusion in milk expression and pumping research and in environmental impact research. This article uses the term pumped rather than expressed milk. Physiologically, economically, and environmentally, pumped milk is different from expressed milk.

Substantial economic costs are involved in the equipment purchased relating to pumping, storing, and feeding mother’s milk, and there are also environmental costs. Opportunities to prevent waste and increase resource efficiency are policy priorities in many areas, though these rarely include the consideration of infant feeding related costs. These environmental costs include the manufacturing and disposal of plastic milk collection sets, sterilization products, and feeding equipment, as well as electricity.

High-tech pumps contain plastic and precious and heavy metals (used for circuit boards and batteries), and the total life cycle environmental costs of producing, using, and disposing of this equipment is of concern. Most of the items are single use or single user items and these ultimately end up as waste. Recycling and Waste Electronic and Electrical Equipment (WEEE) treatment are not sustainable solutions. If incinerators are overly full or poorly maintained they emit toxic gases, and dwindling landfill space compounds the situation of the economic and environmental costs and also detrimental health effects.

Oil based plastic manufacturing is resource intensive, and the resulting waste and end of life plastics may never fully decompose if released to the environment. Plastics used in connection with pumping and using mother’s milk may contain the synthetic chemical Bisphenol A (BPA), and phthalates, which are endocrine disrupters, can leech from the plastic into the milk and may affect the child’s reproductive system and development with intergenerational effects [4,5].

Many pumps are single-user items and may become waste products after a few months when the mother is no longer pumping; some mothers also purchase multiple pumps in an attempt to find a suitable pump. Manufacturers recommend that open loop pumps should not be passed on to another user. Battery operated pumps are common and the used batteries become waste products that need special treatment to avoid ill health effects. Many trees are pulped and processed (with detrimental environmental effects) to provide the marketing materials for pumps that are freely distributed at health worker conferences and baby and trade shows.

Many pumps require electricity for use as well as electricity for the storage of the milk. Electricity may be erratic or of limited supply in some areas, thus increasing health inequalities if there is an expectation of reliance on pumped mothers’ milk. There may also be a maternal energy and nutrient cost if significant volumes of milk above the infants’ needs are pumped only to be thrown out as there are no storage facilities [6].

There are well publicized health risks of obesity for children and mothers. There are risks of obesity resulting from bottle feeding of mother’s milk, and even though these risks are lower than those associated with feeding artificial formula, the risks are still higher than those associated with direct breastfeeding [7]. Obesity may also have an environmental impact. It is speculated that obesity “could have the same implications for world food energy demands as an extra half a billion people living on the earth” [8].

Feeding at the breast has been shown to aid child spacing and reduced family size and thus has an effect on population growth [9]. Population growth has been acknowledged as one of the major environmental challenges facing humanity, especially in countries where access to basic human needs such as shelter, water, food, sanitation, and healthcare is constrained. Research on the child spacing effect related to the expressing or pumping of milk is very limited. One study found an association between increased risk of pregnancy in the first six postnatal months with employed mothers who were expressing milk combined with breastfeeding, compared to the non-employed mothers breastfeeding without expressing milk [10]. No research was found on lactation amenorrhea and delayed ovulation when exclusively pumping and this remains a topic that needs further research [10].

The International Code of Marketing of Breast-Milk Substitutes takes into consideration the World Health Organization (WHO) and UNICEF recommendations of the importance for women to have true and objective choices [11]. However, marketing of pump equipment may compromise or remove objective choices for families, in addition to influencing community attitudes, including the attitudes of health workers and insurance providers towards the use of pumps [12,13]. Some breast pump manufacturers have the capacity to spend substantially more than what health services can spend on parental support, and this can erode the environmental benefits associated with feeding at the breast and, thereby, increase detrimental environmental impacts associated with infant feeding actions.

An ongoing project is examining the waste and environmental aspects of infant feeding [3]. Backcasting methodology was used for a theoretical quantification of the waste and environmental aspects. Backcasting methodology can provide an interdisciplinary framework to develop future visions (or scenarios) and analyze how to achieve these visions and facilitate the consideration of environmental issues such as the effects of infant feeding. The first phase publications of this project focus on infant formula and the later phases and publications will include environmental aspects related to human milk pumping [14].

In order for parents, health workers, and health policy makers to make informed decisions about reliance on pumped mothers milk, they need information on its individual immunological and nutritional aspects, as well as information on the broader and far reaching environmental aspects of reliance on pumped mother’s milk.
